# Predation of the Peach Aphid *Myzus persicae* by the mirid Predator *Macrolophus pygmaeus* on Sweet Peppers: Effect of Prey and Predator Density

**DOI:** 10.3390/insects6020514

**Published:** 2015-06-08

**Authors:** Lara De Backer, Felix L. Wäckers, Frédéric Francis, François J. Verheggen

**Affiliations:** 1 Entomologie fonctionnelle et évolutive, Gembloux Agro-Bio Tech, University of Liege, 2 Passage des Déportés, Gembloux, 5030, Belgium; E-Mails: ldebacker@doct.ulg.ac.be (L.D.B.); frederic.francis@ulg.ac.be (F.F.); 2 Centre for Sustainable Agriculture, Lancaster University, Lancaster, LA1, UK; E-Mail: felix.wackers@biobest.be

**Keywords:** biocontrol, predator density, predation, factitious diet, food supplement, pepper plants

## Abstract

Integrated Pest Management strategies are widely implemented in sweet peppers. Aphid biological control on sweet pepers includes curative applications of parasitoids and generalist predators, but with limited efficiency. *Macrolophus pygmaeus* is a zoophytophagous predator which has been reported to predate on aphids, but has traditionally been used to control other pests, including whiteflies. In this work, we evaluate the effectiveness of *M. pygmaeus* in controlling *Myzus persicae* (Homoptera: Aphididae) by testing different combinations of aphid and predator densities in cage-experiments under greenhouse conditions. The impact of the presence of an alternative factitious prey (*E. kuehniella* eggs) was also investigated. *Macrolophus pygmaeus*, at densities of four individuals/plant, caused rapid decline of newly established aphid populations. When aphid infestations were heavy, the mirid bug reduced the aphid numbers but did not fully eradicate aphid populations. The availability of a factitious prey did not influence *M. pygmaeus* predation on aphids. Based on our data, preventive application of *M. pygmaeus*, along with a supplementary food source , is recommended to control early infestations of aphids.

## 1. Introduction

Integrated Pest Management (IPM) strategies are being increasingly used in greenhouses [1,2]. The demand for non-chemical solutions is driven by consumer demands for low pesticide residues on fruits and vegetables, by the reduction in available active compounds due to more stringent regulations and by the hazard of pest resistance development. IPM strategies based on the release of biocontrol agents are very well implemented in European greenhouses [3,4,5,6,7]. In sweet pepper crops, two key pests, namely *Bemicia tabaci* Gennadius (Homoptera: Aleyrodidae) and *Frankliniella occidentalis* Pergande (Thysanoptera: Thripidae), can be effectively controlled by generalist predatory mites such as *Amblyseius swirskii* Athias-Henriot (Acari: Phytoseiidae) [8]. In southeast Spain, *A. swirskii* and the minute pirate bug *Orius laevigatus* Fieber (Hemiptera: Anthocoridae) are the main biocontrol agents used in sweet pepper production [9]. Aphids are another major pest of sweet peppers, and also cause problems on a wide range of cultivated plants [10,11]. On sweet pepers, the most common aphid pest species include *Myzus persicae* Sulzer (having the highest incidence), *Aphis gossypii* Glover, *Macrosiphum euphorbiae* Thomas, and *Aulacorthum solani* Kaltenbach (Hemiptera: Aphididae) [11]. Specialist hymenopteran parasitoids and the predatory midge *Aphidoletes aphidimyza* Rondani (Diptera: Cecidomyiidae) are the main biocontrol agents used against aphids [12]. With the help of banker plants, *Aphidius colemani* Viereck (Hymenoptera: Braconidae) can also be efficiently used against *M. persicae* [13,14]. To complete the list, several other parasitoids could be used against *M. persicae*, including *Aphelinus asychis* Walker (Hymenoptera: Aphelinidae), *Aphidius matricariae* Haliday (Hymenoptera: Braconidae)*,* and *Aphidius ervi* Haliday (Hymenoptera: Braconidae) [15]. Unfortunately, parasitoids are not always sufficiently effective in controlling aphids and the release of other generalist predators is often needed to improve the control [16,17]. This increases the cost of the biocontrol [18].

While specialist parasitoids and predators cannot establish, nor remain on the crop in absence of pests, zoophytophagous predators can take advantage of resources offered by the plant and thus remain on the crop between infestations or even build up populations prior to the pest infestation [19,20,21,22]. Being zoophytophagous and mass-produced, *Macrolophus pygmaeus* Rambur (Heteroptera: Miridae) (formerly identified as *Macrolophus caliginosus* Wagner [23]) is a valuable candidate for pest control in sweet peppers [18,24,25]. It is able to develop by feeding on sweet peppers [26], alhough it reproduces at lower rates [27]. Unlike *Nesidiocoris tenuis* Reuter (Heteroptera: Miridae), plant feeding by *M. pygmaeus* does not cause damage to the plants, unless they reach extremely high numbers of 50+ individuals per plant [18,28]. Also *M. pygmeus* has proven to be a better predator in controlling *M. persicae* in sweet peppers than *Orius majusculus* and *O. laevigatus*, predators widely used in inoculative biocontrol on sweet pepper [18]. While aphids in general are a good food source for *M. pygmaeus* [26], *M. persicae* in particular enhances *M. pygmaeus* longevity and reproduction rate and is actively searched out as prey.

IPM strategies based on *M. pygmaeus* are well implemented in greenhouses to control pest such as whiteflies and the tomato leafminer. Inundative releases involve a large number of predators and are used for curative control. *Macrolophus pygmaeus* is preferentially release prior to infestation, in inoculative releases, to allow a good and sustainable population establishment [29]. Here, we study the curative and preventive effect of *M. pygmaeus* on *M. persicae* and the effect of a supplementary diet on its control.

## 2. Material and Method

### 2.1. Plant and Insect Rearings

Pepper plants, *Capsicum anuum* L., were grown in a greenhouse (Westerlo, Belgium), at a temperature of 25.4 °C ± 3.4 °C and 40%–60% RH. Supplementary lighting was provided (16L: 8D) using mercury-vapor lights. Plants were 60 cm high and on average had 25 true leaves when used in the trials. All rearing and experiments were conducted under the same environment conditions. *Macrolophus pygmaeus* individuals came from a laboratory culture on tobacco (*Nicotiana tabacum* L.) plants on which they were fed with *Ephestia kuehniella* Zeller (Lepidoptera: Pyralidae) eggs. Peach aphids, *M. persicae,* had been reared for several generations on tobacco plants under the same conditions as above.

### 2.2. Effect of Predator Density

In order to evaluate the predator density needed to control a small introduction of aphids as curative application, sixteen 7.5 m^3^ walk-in cages (2.5 m × 1.5 m × 2 m) each containing four pepper plants were used. Flowers and butts were cut off as they can provide feeding resources for the predator [18,30]. Ten aphids (five winged and five wingless adults) were introduced per cage to mimic an early infestation in a greenhouse. They were placed on one of the four plants, all individuals on a randomly chosen leaf.

Three different densities of *M. pygmaeus* were tested; 16, 32, and 48 predators per cage; and compared to a control (no predator added). Unsexed adult *M. pygmaeus* were gently collected with a respirator from the rearing and were released at the bottom of the cages directly after the aphid introduction. Aphids and aphid predators per cage were counted every second day until all aphids were eaten or until *M. pygmaeus* nymphs appeared.

### 2.3. Predation Efficiency on Large Aphid Colonies

To evaluate the ability of *M. pygmaeus* to control high aphid densities, eight walk-in cages (2.5 m × 1.5 m × 2 m) each containing four pepper plant were used. Fifty aphids were released on the highest fully formed leaf of each pepper plant (200 aphids in total), while concurrently sixteen unsexed adult *M. pygmaeus* were released on the floor of the cage. In control cages 200 aphids were released as above, but without release of predators. Four replicates were made for both treatments. Aphids and aphid predators per cage were counted every second day until all aphids were eaten or *M. pygmaeus* nymphs appeared. The initial density of *M. pygmaeus* was maintained by adding predators when required after each counting.

### 2.4. Impact of Alternative Prey on M. pygmaeus Predation

To evaluate the impact of a alternative prey items on the aphid predation potential of *M. pygmaeus*, we released aphids on plants with established *M. pygmaeus*, obtained through three different treatments: (1) pre-inocculation with 10 *M. pygmaeus* without alternative prey; (2) pre-inocculation with 10 *M. pygmaeus* which had been provided with *E. kuehniella* as alternative prey. Control cages consited of clean plants on which no predators had been introduced. Each treatment was replicated four times, *i.e.* four walk-in cages (2.5 m × 1.5 m × 2 m) each containing four pepper plants. To mimic a preventive application of predatory bugs, predators were pre-released in the experimental cages four weeks before aphid introduction. During this four-week period, predators were fed weekly with 1 g of *E. kuehniella* eggs, poured on the top of the plants, to enhance the population establishment. After four weeks, adult aphids (five winged and five wingless individuals) were added to each of the 12 cages as described in the “predation efficiency on large aphid colonies” trial. In the four replicates of the supplementary food treatment, 1 g *E. kuehniella* eggs were added twice a week ad libitum on the top of the leaves. Aphids and predators per walk-in-cage were counted twice a week.

### 2.5. Statistical Analyses

Aphid numbers were compared using a one-way ANOVA, followed by Tukey’s post hoc tests. Population normality and variance equality were previously verified. All tests were conducted with MINITAB v15 (State College, Centre County, PA, USA).

## 3. Results

### 3.1. Effect of Predator Density on Initially Low Aphid Densities

**Figure 1:**
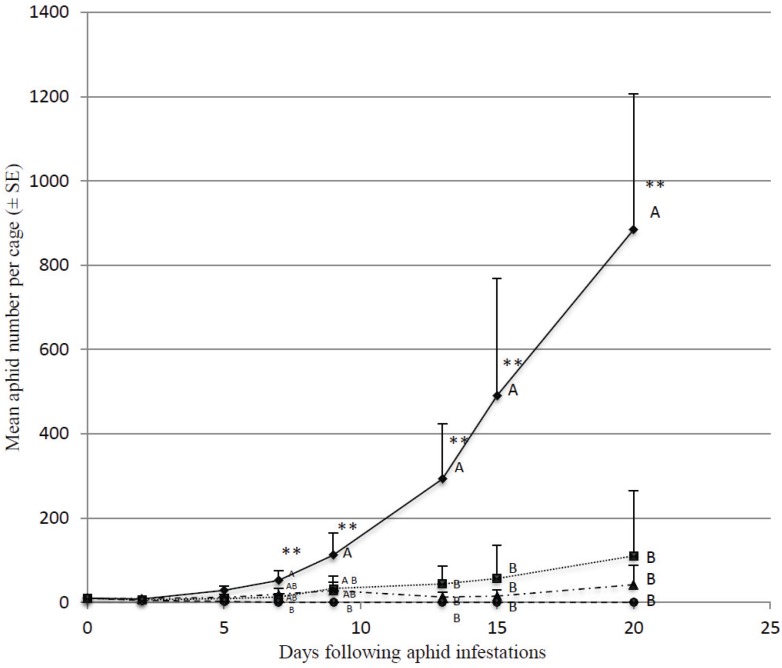
Evolution of mean aphid number in cages (*n* = 4) containing four sweet pepper, in presence of different *M. pygmaeus* densities (0, plain line with diamond shapped dots; 16 per cage, dotted line with square dots; 32 per cage, dashed and dotted line with triangle dots and 48 per cage, dashed line with circle dots).*, stars indicate mean aphid number statistically differing with *M. pygmaeus* density, aphid number sharing the same letter can not be considered as different.

In absence of predators, aphid populations grew rapidly, reaching 885 ± 321 individuals per cage after 20 days (Figure 1). In cages containing 16, 32 and 48 predators, averaged aphid numbers reached 110 ± 109, 42 ± 40 and 0 ± 0, respectively after 20 days. Within 7 days, aphid numbers in the cages with 48 predators were significantly lower than in the control cages (F_3,12_ = 5.33; *p* = 0.014;) and were all eliminated within 9 days. For all tested predator densities, aphid numbers were significantly reduced from the 13th day onwards.

### 3.2. Predation Efficiency on Initially Large Aphid Colonies

**Figure 2:**
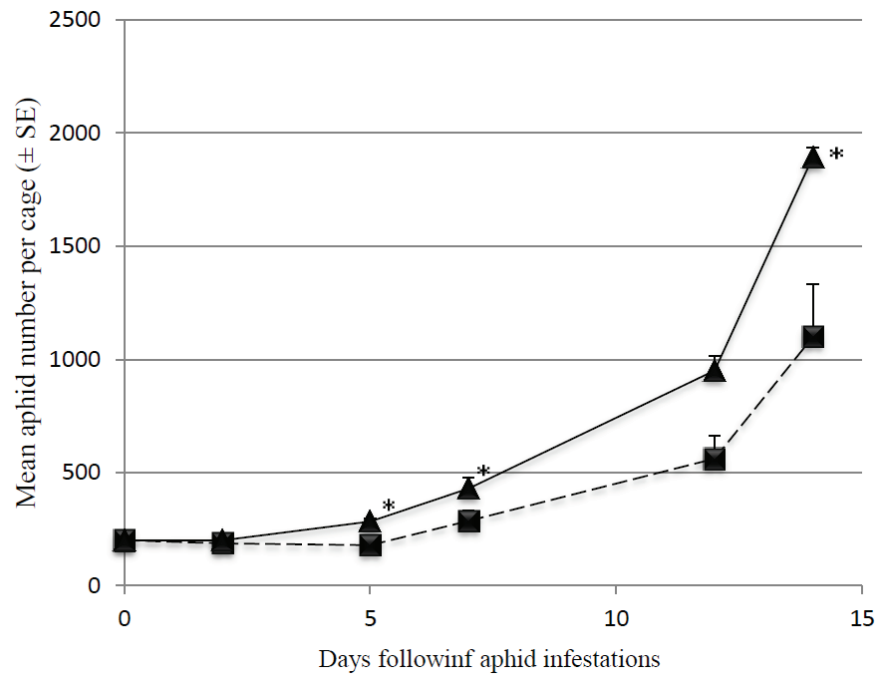
Evolution of mean aphid number (*n* = 4) in presence (dashed line with square dots) and absence (plain line with triangle dots) of *M. pygmaeus*; *, stars indicate mean aphid number statistically differing with *M. pygmaeus* presence.

After 14 days, an average of 1894 ± 41 aphids were present in control cages (Figure 2). Significantly fewer aphids were observed in presence of *M. pygmaeus* after 5 days (F_1,6_ = 19.02; *p* = 0.050). After 14 days, the average aphid number was 42% lower compared to the control cages (1099 ± 235 aphids ; F_1,6_ = 11.05; *p* = 0.016).

### 3.3. Impact of Alternative Prey on M. pygmaeus Predation

**Figure 3:**
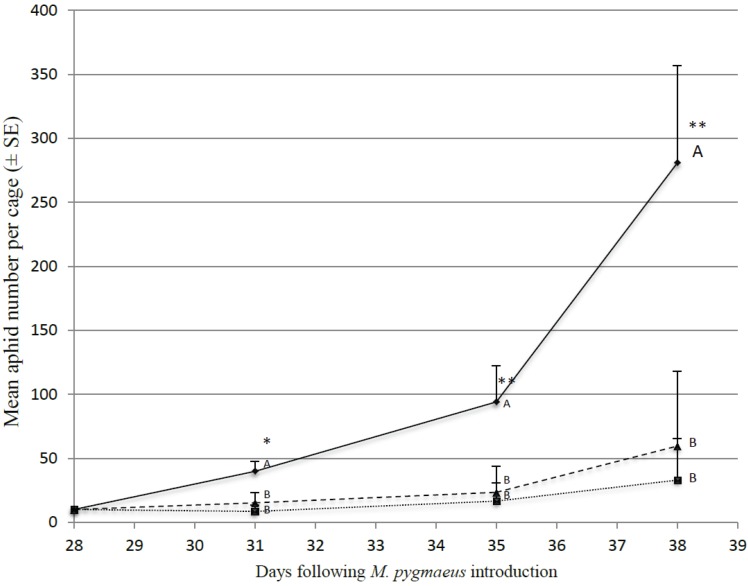
Evolution of mean aphid number per cage (*n* = 4) in absence of predator (plain line), in presence of predator (dotted line and square dots) and in presence of predators and supplementary diet (dashed line with triangle dots).*, stars indicate mean aphid number statistically differing with the treatment; means sharing the same letter can not be considered as different.

Figure 3 shows the aphid population dynamics for the three treatments (control; without alternative diet; with *E. kuehniella* eggs), keeping in mind that predatory bugs were present for 28 days prior to aphid introduction (corresponding to day 0). After 31 days, aphid populations were significantly lower in presence of predators (F_2,9_ = 5.28; *p* = 0.015). At the end of the test (day 38 on Figure 3), the average aphid numbers were 281 ± 76 in the control cages, 33 ± 32 in the cages containing *M. pygmaeus* but without *E. kuehniella* eggs and 59 ± 58 in the cages with both *M. pygmaeus* and *E. kuehniella* eggs (*p* = 0.006; F_2,9_ = 6.8). Tukey test groups both treatments involving predators in the same group apart from the cages without predators.

Twenty-eight days after *M. pygmaeus* release, the average number of predators per cage was 42 ± 5 (*n* = 8). One week after the aphid introduction, the average number of *M. pygmaeus* was significantly higher in the cages containing aphids and *E. kuehniella* eggs (61 ± 7) than in the cages without *E. kuehniella* eggs (39 ± 5) (F_1,6_ = 4. 64; *p* = 0.041).

## 4. Discussion

*Macrolophus pygmaeus* predation behavior on *M. persicae* has been largely investigated using various conditions and plants including sweet peppers [9,24,25,26,27,31]. The effect of plant food on prey consumption has also been documented, pepper flower presence decreased prey consumption [30]. The ability of *M. pygmaeus* to reduce aphid population development is confirmed in every trial conducted here. In previous work, a single *M. pygmaeus* adult has been osberved to consume between 10 and 21 aphids per day under laboratory conditions [24,31,32]. Based on our data, the predation rate seems to be lower under simulated-field conditions. Its phytophagous behaviour might be one of the explanations for this reduced predation rate compared to laboratory assays.

In our first experiment, we evaluated the ability of three different *M. pygmaeus* densities to control newly established small aphid colonies (10 individuals). The lowest density of *M. pygmaeus* tested (16 individuals per 7.5 m^3^ cage) yielded a 88% reduction of the aphid numbers, compared to the control cage (absence of predator) where nearly 900 individuals were observed. However, the aphid population kept increasing. Lykouressis *et al.* [30] evaluated *M. pygmaeus* predation in Petri dishes on small aphid densities. No matter the aphid number offered, on sweet pepper, predators never ate all of them. Even if they were able to eat far more items than offered in small colonies when offered larger ones. *M. pygmaeus* exhibit a Holling’s Type II functionnal response, eating more prey when large amounts are available [33,34]. Only, higher predator densities of 48 *M. pygmeus* per cage could achieve a complete eradication of aphids. Although these predator densities are high for a commercial release, it could be realized by applying these zoophytophagous predators preventively on the crop before aphid infestation. Our data demonstrate the ability of *M. pygmaeus* to protect the crop against new aphid infestations.

In our second experiment, we evaluated the ability of *M. pygmaeus* to control large aphid populations, by applying more aphids, and thus a lower predator/prey ratio than in the first set of experiments (1/12.5 *versus* 1/0.625 to 1/0.21 respectively). We found that, while *M. pygmaeus* reduced aphid population growth, it was not able to suppress aphid populations which kept increasing, ending above the economic injury level. In absence of predators, the initial aphid colony of 200 individuals reached almost 1900 individuals, while this figure was approximately 1000 individuals in presence of predators. Our results contrast with those of Fischer [9] who showed that a couple of predators succeeded in controlling heavy aphid infestations on six sweet peppers infested with 70 aphids per leaf, corresponding to a predator ratio much lower than 1/12.5. However, these authors have observed a reduction in aphid numbers simultaneously in their control populations (absence of predator) and the aphid population controlled with predators. Curative release of *M. pygmaeus* to control aphid populations infesting sweet peppers could therefore be applied, but a ratio predator/pest lower than 1/12.5 seems not sufficient to control heavy infestations (50 aphids per plant). This conclusion is also supported by the results of Messelink *et al.* [24].

The establishment of a predator population is the critical part of the biocontrol using generalist predators [18,22]. Our last trial showed that a four week establishment period with supplementary food is sufficient to double predator numbers and thus realize a population that can control small aphid infestations. Messelink *et al.* [24] previously demonstrated that predators grew a population faster with supplementary food over a 7 week period. These authors suggested that *M. pygmaeus* efficiency to control aphids is increased by the addition of a supplementary food source. Our experiment did not show any significant difference in terms of aphid control between cages with or without *E. kuehniella* eggs. However, during the 4 weeks establishment period, all predators were provided with *E. kuehniella* eggs. This results in similar predator numbers in every cage at the start of the aphid introductions. While Messelink *et al.* [24] started the treatment before the establishment period, so predators studied for their aphid control capacity without supplementary food had to build up their population only feeding on the plants. In that case, at the time of aphid introduction, predator numbers were unequal between the treatments.

It is interesting to note that the elevated predator numbers in the continuous *E. kuehniella* feeding treatment did not translate in better aphid control. If anything, there was a tendency (albeit not significant) for higher aphid numbers in the treatment with continued *E. kuehniella* provision. This implies that *E. kuehniella* feeding when aphids are present, may result in predator satiation effects [22].

Based on our data, preventive application of *M. pygmaeus*, together with a suitable alternative food source, is recommended to control early infestations of aphids. Once the pest is present in the crop, reducing or terminating the supplementation of Ephestia may be the best solution.

## 5. Conclusions

This study aimed to evaluate *M. pygmaeus* predation on *M. persicae* in currentive application on small and large aphid infestations and in preventive application with supplementary food. In every case, predators managed to significantly and drastically reduce aphid numbers. However, aphid populations kept increasing very slowly, except when predators occurred at the amount of 12 individuals per plant and succeeded to eradicate the pest. The availability of a supplementary food did not influence *M. pygmaeus* predation on aphids but allowed a faster establishment of predator populations when released prior to infestation. The establishment period is the key to achieve good control level as large numbers of predators are needed to eradicate the pest. Based on our data, preventive application of *M. pygmaeus*, along with a supplementary food source, is recommended to control early infestations of aphids.
